# Effects of Spike Mutations in SARS-CoV-2 Variants of Concern on Human or Animal ACE2-Mediated Virus Entry and Neutralization

**DOI:** 10.1128/spectrum.01789-21

**Published:** 2022-05-31

**Authors:** Yunjeong Kim, Natasha N. Gaudreault, David A. Meekins, Krishani D. Perera, Dashzeveg Bold, Jessie D. Trujillo, Igor Morozov, Chester D. McDowell, Kyeong-Ok Chang, Juergen A. Richt

**Affiliations:** a Department of Diagnostic Medicine/Pathobiology, College of Veterinary Medicine, Kansas State University, Manhattan, Kansas, USA; University of Georgia

**Keywords:** SARS-CoV-2, variants of concern, spike mutations, virus entry, virus replication, neutralization

## Abstract

Severe acute respiratory syndrome coronavirus 2 (SARS-CoV-2) is a zoonotic agent capable of infecting humans and a wide range of animal species. Over the duration of the pandemic, mutations in the SARS-CoV-2 spike (S) protein have arisen, culminating in the spread of several variants of concern (VOCs) with various degrees of altered virulence, transmissibility, and neutralizing antibody escape. In this study, we used pseudoviruses that express specific SARS-CoV-2 S protein substitutions and cell lines that express angiotensin-converting enzyme 2 (ACE2) from nine different animal species to gain insights into the effects of VOC mutations on viral entry and antibody neutralization capability. All animal ACE2 receptors tested, except mink, support viral cell entry for pseudoviruses expressing the ancestral prototype S at levels comparable to human ACE2. Most single S substitutions did not significantly change virus entry, although 614G and 484K resulted in a decreased efficiency. Conversely, combinatorial VOC substitutions in the S protein were associated with increased entry of pseudoviruses. Neutralizing titers in sera from various animal species were significantly reduced against pseudoviruses expressing the S proteins of Beta, Delta, or Omicron VOCs compared to the parental S protein. Especially, substitutions in the S protein of the Omicron variant significantly reduced the neutralizing titers of the sera. This study reveals important insights into the host range of SARS-CoV-2 and the effect of recently emergent S protein substitutions on viral entry, virus replication, and antibody-mediated viral neutralization.

**IMPORTANCE** The ongoing coronavirus disease 2019 (COVID-19) pandemic, caused by the severe acute respiratory syndrome coronavirus 2 (SARS-CoV-2), continues to have devastating impacts on global health and socioeconomics. The recent emergence of SARS-CoV-2 variants of concern, which contain mutations that can affect the virulence, transmission, and effectiveness of licensed vaccines and therapeutic antibodies, are currently becoming the common strains circulating in humans worldwide. In addition, SARS-CoV-2 has been shown to infect a wide variety of animal species, which could result in additional mutations of the SARS-CoV-2 virus. In this study, we investigate the effect of mutations present in SARS-CoV-2 variants of concern and determine the effects of these mutations on cell entry, virulence, and antibody neutralization activity in humans and a variety of animals that might be susceptible to SARS-CoV-2 infection. This information is essential to understand the effects of important SARS-CoV-2 mutations and to inform public policy to create better strategies to control the COVID-19 pandemic.

## INTRODUCTION

Severe acute respiratory syndrome coronavirus 2 (SARS-CoV-2), the etiological agent of coronavirus disease 2019 (COVID-19), unexpectedly emerged in late 2019 and has spread throughout the world, infecting over 517 million people worldwide and causing over 6.2 million deaths as of May 2022 (https://covid19.who.int/). The zoonotic origin and intermediate hosts of SARS-CoV-2 are still unclear, although bats are considered a likely source based on numerous SARS-CoV-2-related bat coronaviruses found in Southeast Asia (1–3). It is now increasingly apparent that SARS-CoV-2 has the capacity to infect several animal species besides humans, increasing concerns that domestic and wild animals may become secondary reservoirs of the virus (4–6). Outbreaks of SARS-CoV-2 in hundreds of mink farms in the European Union (7), where identification of human-to-mink and mink-to-human virus transmissions (8, 9) as well as mink-associated variants led to the culling of over 20 million minks in Denmark, underscored the importance of identifying and assessing the risks associated with this pandemic for animal and human health (10–13). Other animal species, including cats, dogs, ferrets, hamsters, nonhuman primates, white-tailed deer, mice, cattle, pigs, tree shrews, rabbits, raccoon dogs, and fruit bats, have been investigated for their susceptibility to SARS-CoV-2 infection (14). Reports from natural and experimental infection studies determined a wide range of susceptibility in several domesticated (farm or companion) animals or wildlife to SARS-CoV-2 infection, including white-tailed deer (7, 8, 15–24) (https://www.oie.int/en/what-we-offer/emergency-and-resilience/covid-19/#ui-id-3).

SARS-CoV-2 is an enveloped, positive-sense RNA virus that belongs to the family *Coronaviridae.* RNA viruses are prone to high mutation rates, giving rise to new variants, although the mutation rate of coronaviruses is lower than that of many other RNA viruses due to proofreading activity of their replicative complex (25, 26). Some virus variants possess notable changes in virus transmissibility, virulence, or other characteristics that are important in host defense, such as immune evasion. Since the emergence of COVID-19, multiple variants of SARS-CoV-2 have been identified and have largely replaced the prototype SARS-CoV-2 strain (Wuhan-Hu-1) (27, 28). Currently, the World Health Organization designated Alpha (lineage B.1.1.7), Beta (B.1.351, B.1.351.2, and B.1.351.3), Gamma (P.1, P.1.1, and P.1.2), Delta (B.1.617.2, AY.1, and AY.2), and Omicron (B.1.1.529) SARS-CoV-2 viruses as variants of concern (VOCs) (18, 29), as they are associated with increased risks to global public health. These variants contain multiple amino acid substitutions in the spike (S) protein, some of which have received special attention as they span the receptor-binding domain (RBD) or the S1/S2 junction. Entry of SARS-CoV-2 to the target cells is mediated by the interaction of the S protein with its receptor angiotensin-converting enzyme 2 (ACE2) on the host cell membrane (2, 30, 31). The RBD in the S protein is located on residues 319 to 541 and interacts with 25 conserved residues on human ACE2 (hACE2) (31, 32). Cleavage of the S1/S2 junction (residues 613 to 705) of SARS-CoV-2 S protein by cellular proteases triggers fusion and viral entry into host cells (33, 34). Due to its involvement in receptor binding, most neutralizing antibodies are directed against the RBD (35). Mutations affecting the S protein, including the RBD, are of particular concern because they may enhance virus transmissibility and reduce neutralizing antibody binding and immune protection, thus compromising vaccine and therapeutic antibody efficacies (27). In addition, the interaction between the cellular receptor and virus, leading to virus entry into host cells, is one of the critical factors that determine host susceptibility to virus infection. With the recently emerged virus variants, it is also critical to understand the impact and significance of such mutations on virus neutralization, which has wide-reaching implications on vaccine efficacy; and on animal susceptibility to SARS-CoV-2 in order to identify and manage risks of zoonotic/reverse zoonotic infections. Some of the key mutations found in SARS-CoV-2 VOCs have been studied using pseudotyped viruses or recombinant viruses carrying mutant SARS-CoV-2 S proteins (36); however, only limited information on the role of these mutations for a broad range of animal species, as well as humans, is available so far.

Small animal models, such as mice and Syrian Golden hamsters, are available to study various aspects of SARS-CoV-2 infection and pathogenesis (37). Parental (Wuhan-like) SARS-CoV-2 viruses can infect genetically engineered mice that express hACE2, although unmodified mice are only permissive to mouse-adapted SARS-CoV-2 (38, 39), with the exception of SARS-CoV-2 variants containing the N501Y polymorphism in their S protein (40). Hamsters are highly permissive to SARS-CoV-2 infection, and efficient virus replication and moderate to severe lung pathology are observed following virus replication, usually accompanied by weight loss and other clinical signs during acute infection (41–44). Small animal models for COVID-19 have been used to study viral transmission, pathogenesis, and immunity as well as to evaluate vaccines and therapeutic drugs and are also suitable models for investigating virulence and infectivity of SARS-CoV-2 variants (45).

In this study, we investigated the characteristics of key mutations found in Alpha, Beta, Gamma, and Delta VOCs (single or combinations of 614G, 501Y, 484K, 452R, and 478K mutations). Using lentivirus-based pseudotyped virus assays, the effects of key substitutions on virus entry into human and various animal ACE2-expressing cells and on the neutralizing activities of antisera from humans, cats, and rabbits were determined. In addition, we generated key substitutions (501Y, 484A, 417N, 446S, 440K, 477N, 478K, 493R, and 498R) found in the Omicron VOC and examined the effects of these substitutions on the neutralizing activities of the respective antisera. Using the hamster model, infection studies were conducted to provide further understanding of the replication capacity and pathogenicity of SARS-CoV-2 variants. Finally, structural models for the parental and mutant SARS-CoV-2 RBDs in complex with ACE2 from various animals were generated to probe the structural basis for host susceptibility and the effects of the mutations on the interactions between ACE2 and RBD. The presented results provide important insights into the impact of S protein mutations found in emerging SARS-CoV-2 variants on cell entry in human and other animal species and on virus replication and virus neutralization

## RESULTS

### Entry of pseudotyped virus with SARS-CoV-2 S into HEK293T or Crandell-Rees feline kidney (CRFK) cells expressing human or animal ACE2.

**FIG 1:**
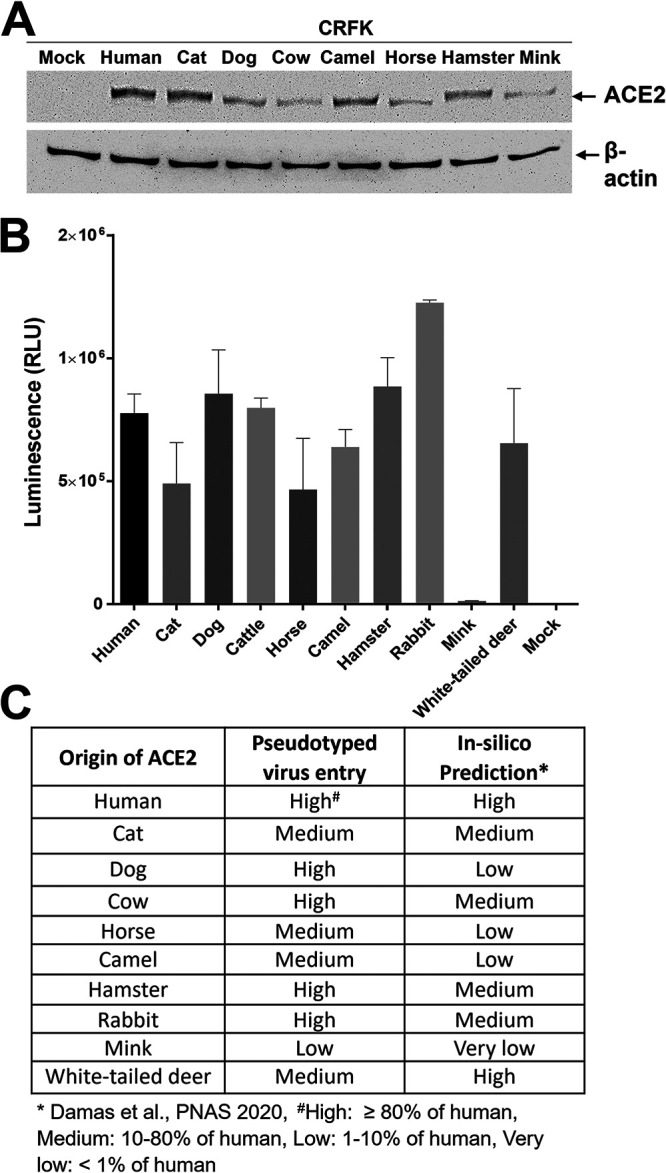
Effects of various ACE2 constructs on the entry of pseudotyped viruses carrying SARS-CoV-2 S into CRFK cells stably expressing ACE2 from various animal species. (A) Western blot of CRFK cells stably expressing various ACE2 receptors or mock cells (no ACE2 transfection). Cell lysates were collected and probed using anti-ACE2 receptor or β-actin antibodies. (B) CRFK cells stably expressing various ACE2 receptors or mock cells (no ACE2 transfection) were infected with pseudotyped virus carrying the parental SARS-CoV-2 S protein. Following incubation of the cells with the pseudotyped virus for 48 h, cells were lysed, and luminescence units were measured. Each bar indicates the mean and the standard error of the means. (C) Summary of the results from the pseudotyped virus entry assay in B. Virus entry levels were considered high, medium, or low when greater than 80%, 10 to 80%, or 1 to 10% of virus entry in ACE2-expressing cells (compared to human ACE2 cells) was observed, respectively, based on the criteria suggested by Damas et al. (17). The asterisk (*) indicates *in silico* predictions by Damas et al. (17).

Expression of ACE2 in human kidney-derived HEK293T or CRFK cells that were stably transfected with a plasmid encoding the ACE2 protein from humans and various animal species was confirmed by Western blotting (Fig. 1A). Entry of pseudotyped viruses, measured by firefly luciferase, was comparable between HEK293T and CRFK cells expressing the same ACE2 construct. However, CRFK cells yielded more robust and consistent results than HEK293T cells; therefore, CRFK cells were subsequently used for pseudotyped virus entry assays. The results of the virus entry assays are shown in Fig. 1B and C. Importantly, native CRFK cells that do not express exogenous ACE2, only inherent feline ACE2 (mock), yielded negligible virus entry (Fig. 1B), indicating that CRFK cells are suitable to determine the effects of exogenous heterologous ACE2 on viral entry. Expression of various animal ACE2 receptors in CRFK cells led to greatly enhanced entry of pseudotyped viruses expressing the parental SARS-CoV-2 S protein (Fig. 1B), except for mink ACE2, which did not show the marked increase in virus entry compared to the other animal ACE2s; however, mink ACE2 had a 31-fold increase over nontransfected cells. Cellular entry of pseudotyped viruses in the presence of ACE2 receptors from various animal species ranged from an approximately 1,200-fold (horse/cat) to 3,000-fold (rabbit) increase in cellular entry compared to the mock control (no ACE2 transfection). Figure 1C shows a summary of the virus entry results using cells expressing different animal ACE2 receptors compared to cells expressing human ACE2. Virus entry levels for each ACE2 species were considered high, medium, or low when greater than 80%, 10 to 80%, or 1 to 10% of virus entry in ACE2-expressing cells (compared to hACE2-expressing cells) was observed, respectively, based on the criteria suggested by Damas et al. (17). High levels of virus entry were observed in cells expressing ACE2 from human, dog, cow, hamster, or rabbit (Fig. 1B and C), while medium levels of virus entry were seen in cells expressing ACE2 from cat, horse, camel, and white-tailed deer. Expression of mink ACE2 resulted in low virus entry. The overall trend of virus entry in cells expressing various animal ACE2 receptors was similar to the *in silico* predictions by Damas et al. (17) (Fig. 1C).

### Entry of pseudotyped virus expressing SARS-CoV-2 parental or mutant S in human ACE2-expressing CRFK cells.

**FIG 2:**
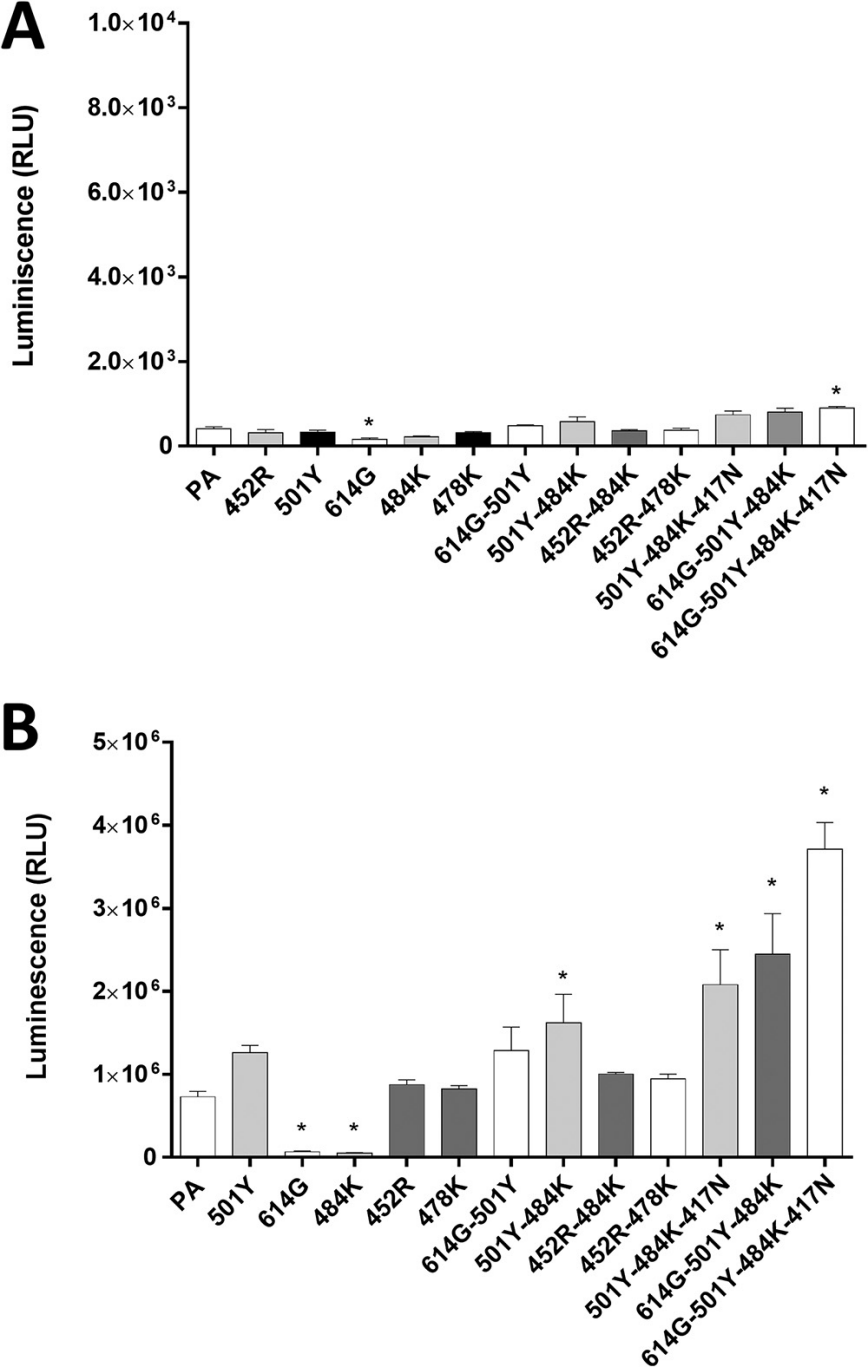
Entry of pseudotyped viruses carrying SARS-CoV-2 S with single or multiple substitutions on the RBD site into nontransfected CRFK or CRFK cells stably expressing human ACE2. No human ACE2-expressing CRFK cells (A) or human ACE2-expressing CRFK cells (B) were infected with pseudotyped viruses with single or multiple RBD substitutions. Following incubation of the cells for 48 h, luminescence units were measured. Each bar indicates the mean and the standard error of the means. PA indicates parental pseudotyped virus (no mutation in the S protein). One-way ANOVAs on the log_10_-transformed raw relative luminescence units were used to compare the parental (PA) group and other groups. Statistical differences between mutation and the parental virus groups are indicated with an asterisk (*, *P* < 0.05).

The pseudotyped virus preparations carrying single or multiple amino acid substitutions in S were quantitated and normalized by enzyme-linked immunosorbent assay (ELISA) p24 lentivirus antigen measurement or by SARS-CoV-2 S protein expression after transduction of the cells. Virus entry of each pseudotyped virus carrying single or multiple substitutions of 417N, 452R, 478K, 484K, 501Y, or 614G on the RBD of the S protein was compared to that of parental pseudotyped viruses (no substitution in S gene) in cells expressing human ACE2 or native CRFK cells (no human ACE2 expression). In CRFK cells expressing no exogenous ACEs (native feline ACE2-expressing CRFK cells), a significant decrease or increase in pseudotyped virus entry was observed with the 614G single mutation or the 614G-501Y-484K-417N quadruple mutation, respectively (Fig. 2A). However, the overall magnitude of pseudotyped virus entry in nontransfected CRFK cells was very low regardless of the presence or absence of S protein mutations, which confirms that nontransfected CRFK cells are poorly supportive of SARS-CoV-2 S-pseudotyped virus entry. However, expression of human ACE2 markedly enhanced viral entry compared to native CRFK cells (Fig. 2B). In these cells, single substitutions of 501Y, 452R, or 478K did not lead to a statistically significant difference in virus entry compared to parental virus (Fig. 2B) except for 614G or 484K, which showed significantly reduced virus entry compared to the parental pseudotyped virus. Among the double substitutions (i.e., 614G-501Y, 501Y-484K, 452R-484K, or 452R-478K), only the 501Y-484K combination significantly increased pseudotyped virus entry compared to the parental pseudotyped virus. The addition of substitution 417N or 614G to the 501Y-484K combination, however, did not further increase the virus entry efficiency of pseudotyped virus compared to the 501Y-484K double substitution unless both 417N and 614G were combined with 501Y-484K in a quadruple combination (417N-484K-501Y-614G). Interestingly, when 501Y was combined with 614G (614G-501Y double substitution), an increase of virus entry was observed similar to the level of parental virus and the single 501Y virus. Virus entry capacity was further enhanced by the addition of 484K (614G-501Y-484K) or 484K-417N (614G-501Y-484K-417N). Similarly, the combination of 501Y and 484K led to significantly increased virus entry compared to the parental virus, suggesting that the 501Y substitution is important in negating the suppressive effects of the 484K and 614G single mutations (Fig. 2B). The reduced virus entry due to the 484K substitution was also restored to the level of the parental virus entry when combined with the 452R substitution (Fig. 2B). However, the 452R-478K double mutation did not lead to enhanced virus entry compared to the 452R or 478K single mutations.

### Entry of pseudotyped virus carrying SARS-CoV-2 parental or mutant S proteins in various ACE2-expressing CRFK cells.

**FIG 3:**
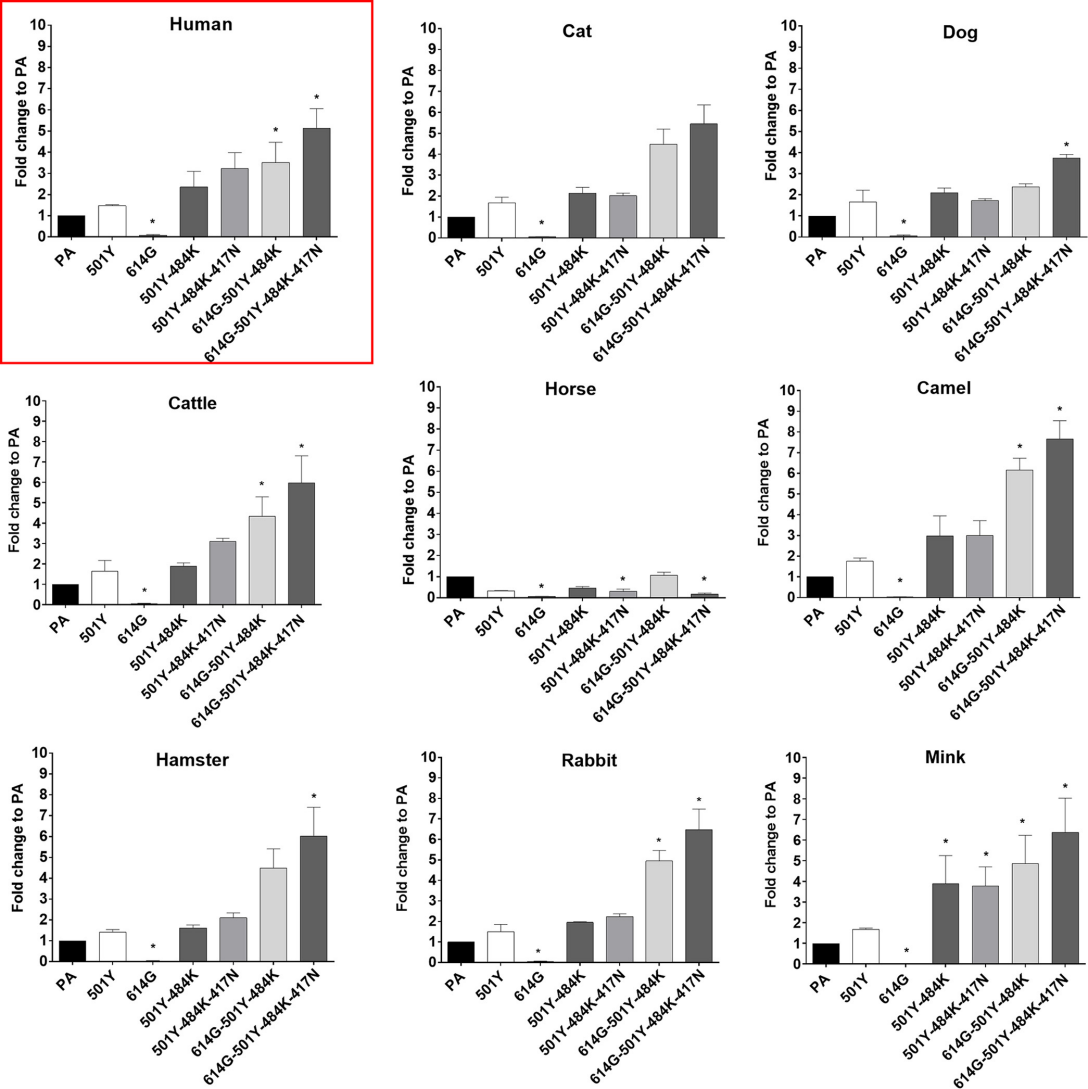
Entry of pseudotyped viruses carrying SARS-CoV-2 S with single or multiple mutations on the RBD site of S protein into CRFK cells expressing ACE2 of various species. CRFK cells expressing ACE2 from different animal species were infected with pseudotyped viruses expressing single or multiple S protein substitutions. Following incubation of the cells for 48 h, cells were lysed, and relative luminescence units were measured. Each mutant pseudotyped virus was compared with the parental pseudotyped virus (PA), and data are presented as the fold change to PA. One-way ANOVAs on the log_10_-transformed raw relative fluorescence units were used to compare the parental group and other groups. Statistical differences between mutation and the parental virus groups are indicated with an asterisk (*, *P* < 0.05). Red square: human data.

In this experiment, we compared the entry of pseudotyped viruses with parental or mutant S into cells expressing ACE2 from various animal species, including humans. Overall, the trend of change in virus entry among various pseudotyped viruses was similar in all tested cells expressing various animal ACE2 receptors (Fig. 3). In general, the quadruple 614G-501Y-484K-417N substitution showed the highest fold increase compared to the parental S (no mutation), followed by the triple combination 614G-501Y-484K. The 501Y-484K and 501Y-484K-417N substitutions led to moderately increased virus entry compared to the parental S but without a statistically significant difference. The 614G single mutation led to a decrease in virus entry in cells expressing human and animal ACE2 (Fig. 2A and Fig. 3). Notably, even in mink ACE2-expressing cells, which support limited virus entry compared to other ACE2s, a similar trend was observed with pseudotyped viruses with single and multiple substitutions (Fig. 3). Interestingly, relatively little change was observed in virus entry among parental and mutant pseudotyped viruses in horse ACE2-expressing cells (Fig. 3). These results suggest that the effects of these mutations in the RBD region of the S protein for virus entry are shared among a wide range of animal ACE2 receptors.

### Structural modeling insights into the ACE2-RBD interaction of different species.

**FIG 4:**
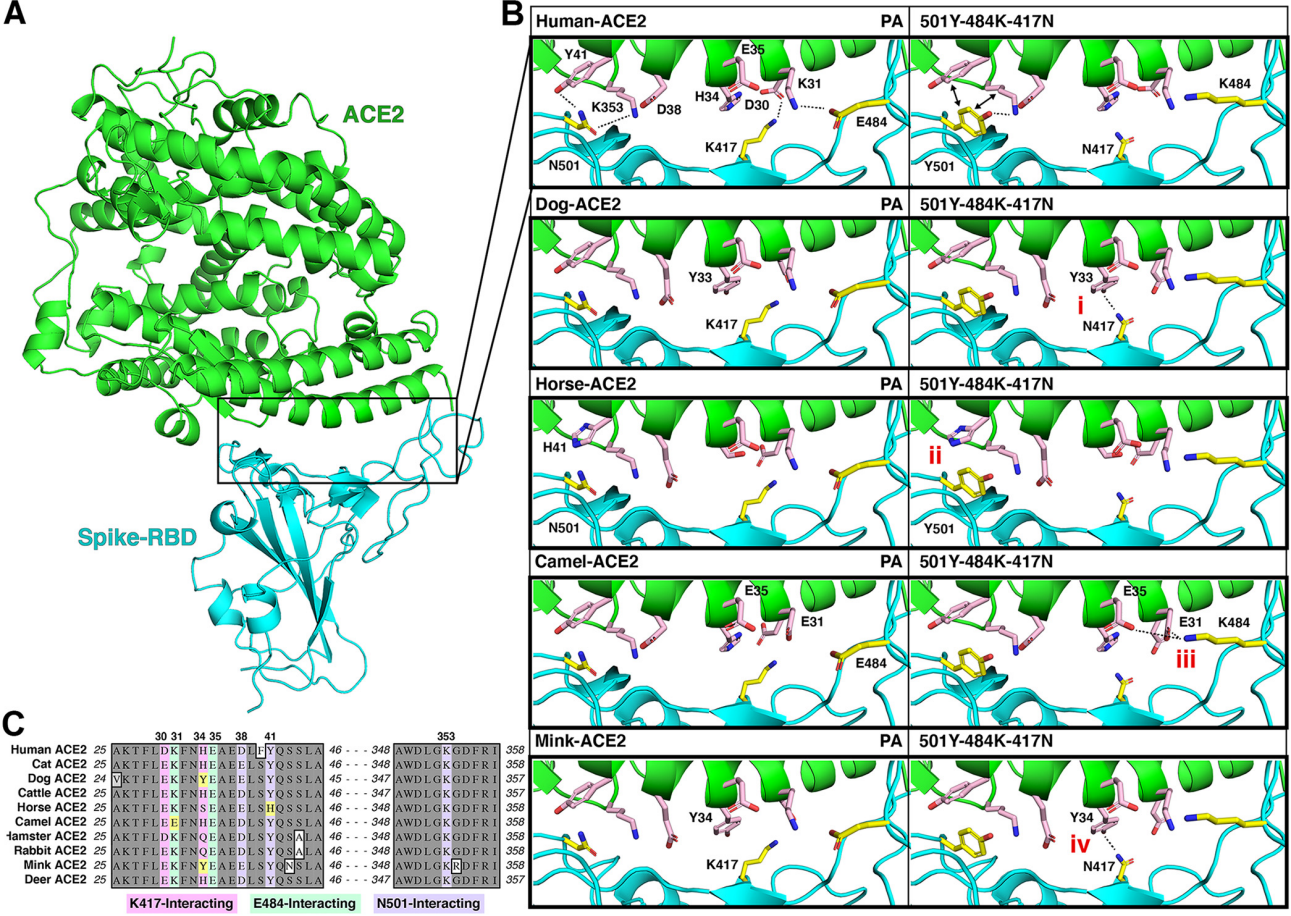
Structural modeling insights into the ACE2-RBD interaction in different species. (A) Illustration of the human ACE2 (green) interaction with the SARS-CoV-2 RBD (cyan) as determined by Protein Data Bank (PDB) entry 6GLZ (93). The inset outlines the location of the ACE2-RBD interface illustrated in C. (B) Summary of significant differences from the human ACE2-RBD interface in the investigated species, highlighting notable differences in (i) dog, (ii) horse, (iii) camel, and (iv) mink. (C) Alignment of the ACE2 amino acid sequences of each species, with K417 (pink), E484 (green), and N501 (purple) interacting residues highlighted. Significant differences outlined in B are highlighted in yellow.

Structural modeling (Fig. 4A) showed that parental SARS-CoV-2 RBD forms hydrogen-bonding interactions (dotted lines) at the following RBD positions: (i) N501 with human ACE2-Y41 (hACE2-Y41) and hACE2-K353, (ii) K417 with hACE2-D30, and (iii) E484 with hACE2-K31 (Fig. 4B). In the triple 501Y-484K-417N mutant, Y501 interacts with hACE2-Y41 and hACE2-K353, and the salt bridges between N417 and K484 and the ACE2 receptor are lost (Fig. 4B). No significant structural differences in the RBD-ACE2 interactions were observed for the PA or mutated RBD for ACE2 from cat, cattle, hamster, rabbit, or white-tailed deer (data not shown). However, dog and mink ACE2 receptors contain an ACE2-H33Y/H34Y substitution, respectively (Fig. 4C) that may increase binding affinity in the presence of the S protein K417N substitution due to potential interactions between the hydroxyl group of tyrosine and the amino group of asparagine (Fig. 4B). Moreover, horse ACE2 contains a Y41H substitution (Fig. 4C) that does not provide the interactions necessary for strong binding of either the PA N501 or mutant Y501 residues (Fig. 4B). Lastly, camel ACE2 contains a K31E substitution (Fig. 4C), which may provide a basis for polar contacts in the region in the presence of the S protein K484 substitution. Overall, analysis of these structural models provides insights into the cell entry of the parental and mutated S proteins in the presence of ACE2 receptors from different animal species.

### Replication and infection dynamics of SARS-CoV-2 variant strains.

**FIG 5:**
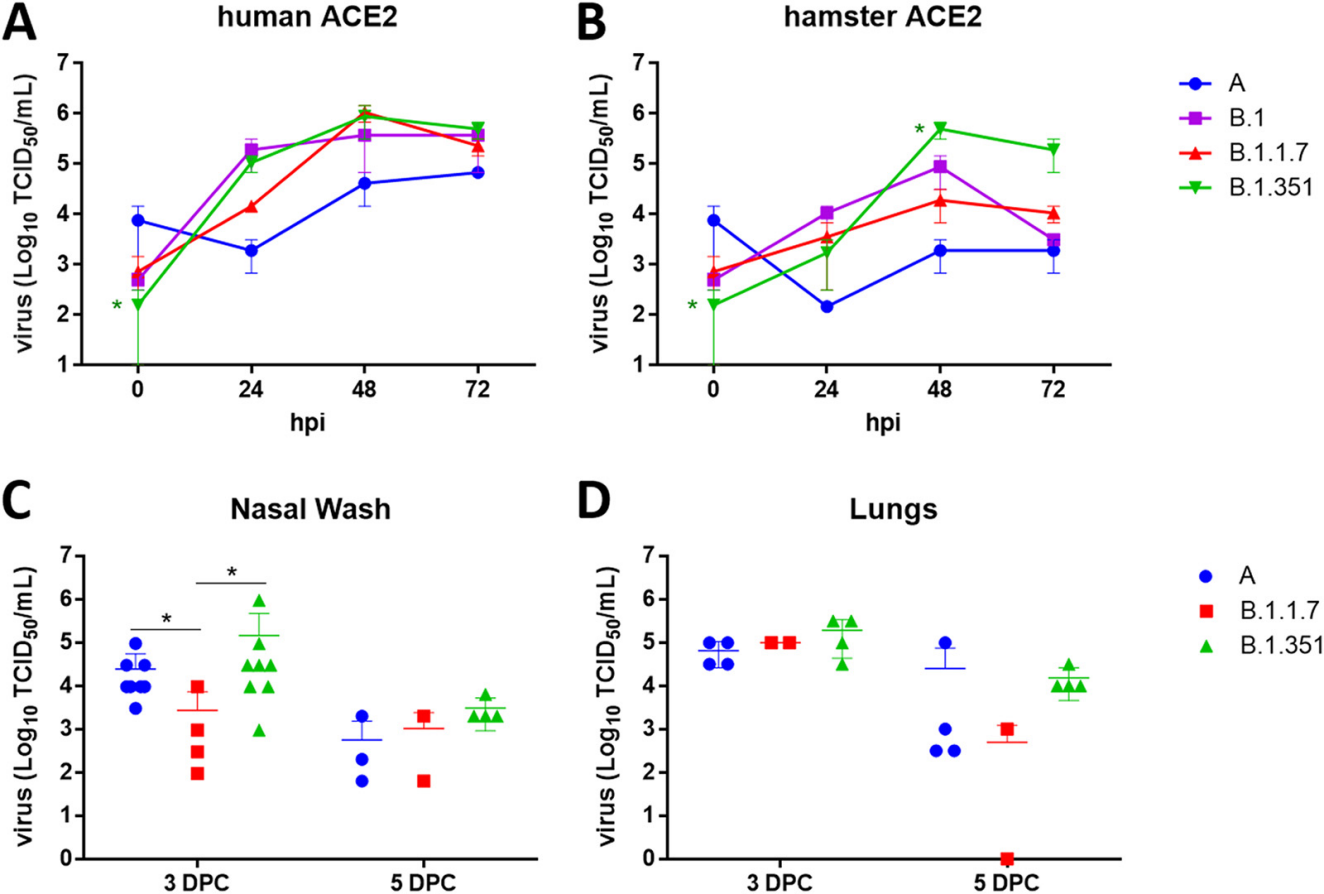
Replication and infection dynamics of SARS-CoV-2 variant strains in cells and hamsters. Replication kinetics were performed in CRFK-human ACE2 (A) or CRFK-hamster ACE2 (B) cells. Virus titrations were performed on infected cell culture supernatants collected at 24, 48, and 72 h postinfection (hpi) and the virus inoculum (time point 0). Virus titrations were also performed on the nasal wash (C) and lungs (D) collected from hamsters, inoculated intranasally with 1 × 10^5^ TCID_50_ of each virus strain, at 3 and 5 days postchallenge (dpc). Mean and standard deviations are shown for each data set. A two-way ANOVA of transformed virus titer data was performed, and statistical differences of the variants compared between each of the strains per time point are indicated by an asterisk (*, *P* < 0.05).

To gain insights into the consequences of VOC-specific RBD substitutions, replication kinetics of parental lineage A (WA1/2020), B.1, B.1.1.7 (Alpha), and B.1.351 (Beta) SARS-CoV-2 strains in CRFK-human ACE2 or CRFK-hamster ACE2 cells were determined (Fig. 5A and B). Although a 0.01 multiplicity of infection (MOI) for all viruses was intended, back titration of the inoculum, represented as time zero, showed the B.1.351 strain at a significantly lower input titer (approximately 0.001 MOI) than the lineage A parental prototype strain. However, titers for the lineage A virus were lower at 24, 48, and 72 h postinfection (hpi) than those observed for the B.1, B.1.1.7, and B.1.351 strains for both CRFK-human ACE2 or CRFK-hamster ACE2 cells. Moreover, the B.1.351 titers were higher than the parental lineage A virus in CRFK-human ACE2 cells at 24, 48, and 72 hpi, and B.1.351 replicated more efficiently than the other viruses in the CRFK-hamster ACE2 cells at 48 and 72 hpi. In both cell lines, the B.1 strain replicated the most efficiently at 24 hpi. The B.1, B.1.1.7, and B.1.351 strains had relatively similar titers in the human ACE2 cells at 48 and 72 hpi, while more variation was observed between these strains at the same time points in CRFK-hamster ACE2 cells. All strains peaked at around 48 hpi in both cell lines, although generally lower titers were observed at all time points for CRFK-hamster ACE2 cells than for CRFK-human ACE2 cells. Taken together, these results show that the variant viruses have a replication advantage over the parental lineage A strain in these cells.

Replication capabilities of the parental lineage A, Alpha VOC B.1.1.7, and Beta VOC B.1.351 strains were analyzed in hamsters inoculated intranasally with 1 × 10^5^ 50% tissue culture infective dose (TCID_50_) of the respective viruses (Fig. 5C and D). Mean virus titers of nasal washes collected from the lineage A- and Beta VOC B.1.351-infected groups were significantly higher than those of the B.1.1.7-infected group at 3 days postchallenge (dpc) but not significantly different at 5 dpc. Mean virus titers of lung homogenates were similar for all groups at 3 and 5 dpc, although the B.1.351-infected individuals had generally higher nasal wash and lung titers than the linage A- and B.1.1.7-infected animals.

### Neutralizing activities of convalescent human sera or postinfection/vaccination sera from cat and rabbit against pseudotyped viruses with single S protein mutations.

**FIG 6:**
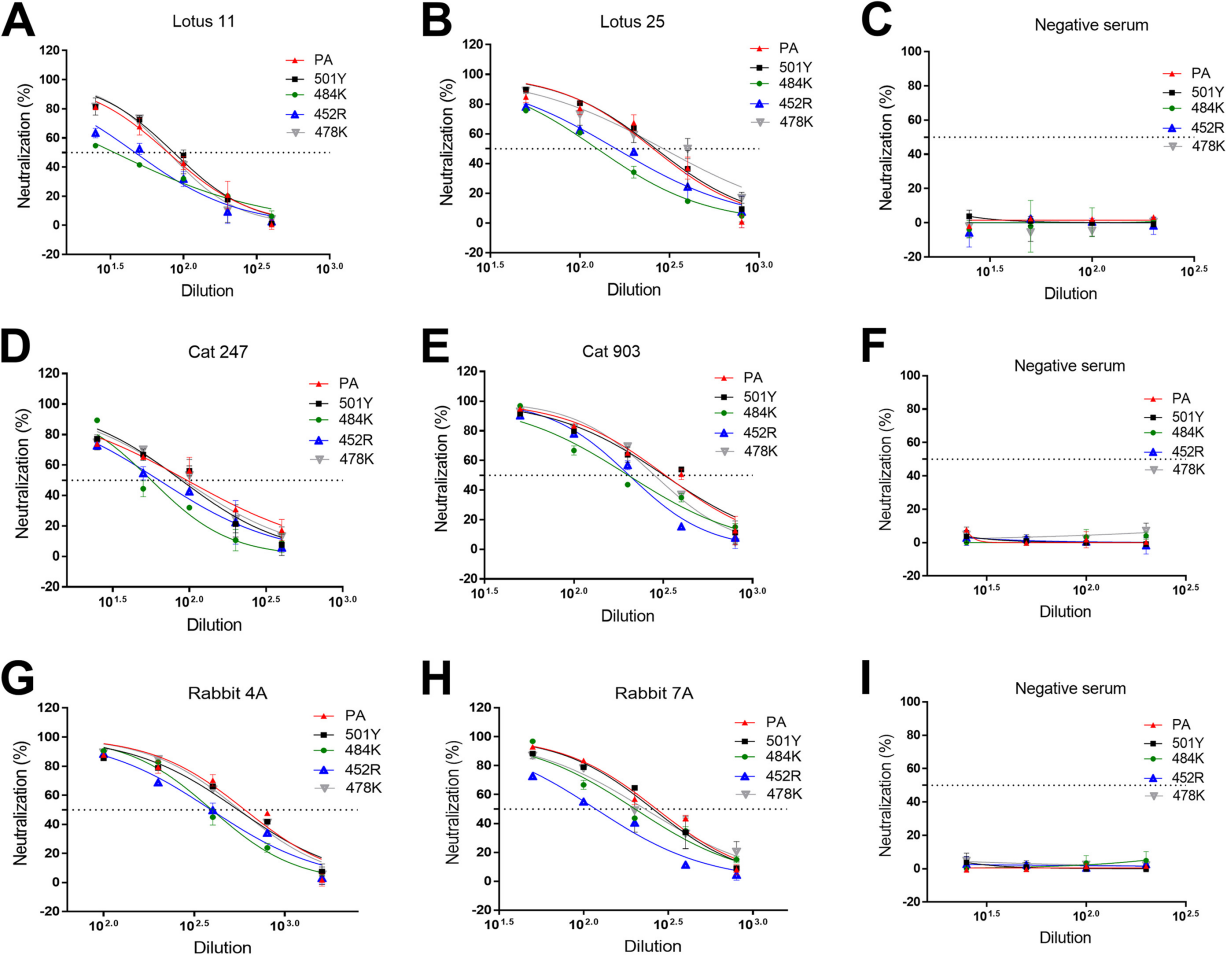
Neutralizing activity of various human, cat, and rabbit sera against pseudotyped viruses carrying SARS-CoV-2 parental or single mutant S using cells expressing human ACE2. CRFK cells expressing human ACE2 were infected with pseudotyped viruses and tested for neutralizing activity against various human (A, B, and C), cat (D, E, and F), and rabbit (G, H, and I) sera. Following incubation of the cells for 48 h, cells were lysed, and the relative luminescence units were measured. Each bar indicates the mean and the standard error of the means. PA indicates parental pseudotyped virus.

The effects of single mutations on the S protein in pseudotyped viruses in the neutralizing activity of various sera from human, cat, and rabbit were assessed using human ACE2-expressing cells. The results are shown in Fig. 6 and Table 1. The negative-control sera from human, cat, and rabbit did not neutralize any of the pseudotyped viruses (starting at a 1:12.5 dilution), as expected (Fig. 6C, F, and I). The 501Y or 478K single substitutions did not affect the neutralizing activity of all tested sera (Fig. 6A to I and Table 1). However, significantly reduced neutralizing activities of human, rabbit, and cat sera were observed against pseudotyped viruses carrying 484K or 452R single substitutions. In contrast, the reduction in neutralization due to the 484K single mutation was not apparent in rabbit 7A serum.

**TABLE 1:** Neutralizing antibody titers of various human, cat, and rabbit sera against pseudotyped viruses carrying SARS-CoV-2 parental (PA) or mutant S proteins tested with human ACE2-expressing cells

Spike mutations	Humans^*a*^			Cats^*a*^			Rabbits^*a*^		
	Lotus 11	Lotus 25	Neg	No. 247	No. 903	Neg	No. 4A	No. 7A	Neg
PA	78.53 ± 2.3	275.65 ± 6.3	<12.5	96.53 ± 4.2	328.60 ± 21.1	<12.5	534.05 ± 77.6	270.10 ± 16.7	<12.5
501Y	85.71 ± 5.3	271.05 ± 34.3	<12.5	89.32 ± 5.9	322.05 ± 30.1	<12.5	550.95 ± 16.4	259.05 ± 22.6	<12.5
484K	33.56 ± 0.5^*b*^	125.00 ± 4.3^*b*^	<12.5	56.82 ± 4.3^*b*^	201.40 ± 2.3^*b*^	<12.5	395.30 ± 22.7	201.40 ± 2.3	<12.5
452R	47.11 ± 5.3^*b*^	161.25 ± 12.7^*b*^	<12.5	64.18 ± 6.9^*b*^	204.15 ± 4.1^*b*^	<12.5	391.65 ± 17.7	117.75 ± 0.8^*b*^	<12.5
478K	78.73 ± 0.3	290.85 ± 14.9	<12.5	92.37 ± 3.4	282.35 ± 5.8	<12.5	563.40 ± 14.5	239.55 ± 55.0	<12.5
501Y-484K	34.38 ± 2.3^*b*^	84.98 ± 2.3^*b*^	<12.5	29.16 ± 0.6^*b*^	135.60 ± 16.7^*b*^	<12.5	265.10 ± 30.7^*b*^	95.06 ± 10.4^*b*^	<12.5
452R-484K	25.62 ± 4.0^*b*^	114.02 ± 23.2^*b*^	<12.5	37.19 ± 0.6^*b*^	143.45 ± 9.4^*b*^	<12.5	201.85 ± 9.2^*b*^	111.10 ± 3.0^*b*^	<12.5
452R-478K	27.40 ± 0.6^*b*^	89.57 ± 5.2^*b*^	<12.5	39.63 ± 7.9^*b*^	103.34 ± 9.9^*b*^	<12.5	228.20 ± 12.9^*b*^	97.63 ± 10.5^*b*^	<12.5
501Y-484K-417N	25.9 ± 0.1^*b*^	90.22 ± 1.6^*b*^	<12.5	31.98 ± 2.0^*b*^	103.20 ± 3.0^*b*^	<12.5	197.05 ± 3.5^*b*^	110.50 ± 4.9^*b*^	<12.5
501Y-484A-417N-446S-440K-477N-478K-493R-498R	18.25 ± 1.95^*b*^	81.76 ± 7.33^*b*^	<12.5	20.86 ± 2.43^*b*^	62.98 ± 13.11^*b*^	<12.5	187.90 ± 12.1^*b*^	70.45 ± 8.48^*b*^	<12.5

a A one-way ANOVA on the neutralizing titers was used to compare the parental and mutant groups. The numbers indicate means and the standard errors of the means. Neg, negative control.

b *P* < 0.05.

### Neutralizing activities of convalescent or postinfection sera from human, cat, and rabbit against pseudotyped viruses with double, triple, or multiple S protein mutations observed in VOCs.

**FIG 7:**
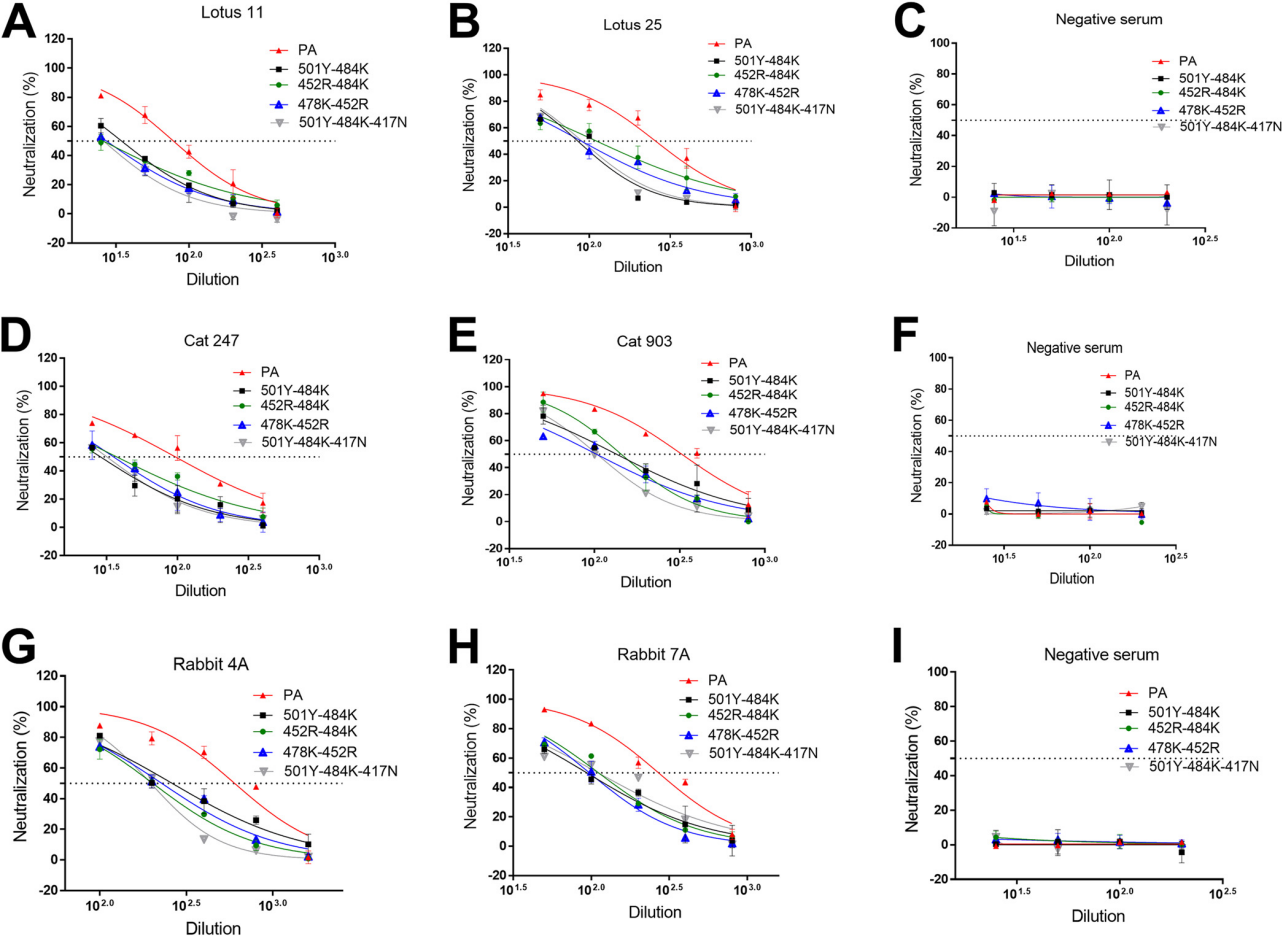
Neutralizing activity of various human, cat, and rabbit sera against pseudotyped viruses carrying SARS-CoV-2 parental (PA) or multiple S mutations using cells expressing human ACE2. CRFK cells expressing human ACE2 were infected with pseudotyped viruses and tested for neutralizing activity against various human (A, B, and C), cat (D, E, and F), and rabbit (G, H, and I) sera. Following incubation of the cells for 48 h, cells were lysed, and the relative fluorescence units were measured. Each bar indicates the mean and the standard error of the means. PA indicates parental pseudotyped virus.

The results of the virus neutralization assays (VNAs) are summarized in Fig. 7 and Table 1. Interestingly, when the 501Y mutation or 478K mutation, which did not affect neutralization as a single substitution (Fig. 6), is combined with 484K or 452R (i.e., 501Y-484K, 452R-478K), neutralizing activities of all tested sera were significantly decreased compared to the parental pseudotyped virus (Fig. 7). Moreover, the double substitution 452R-484K and the triple substitution 501Y-484K-417N also resulted in significant reduction of neutralizing activities of all tested sera compared to the parental pseudotyped virus (Fig. 7). Each of these double and triple substitutions showed comparable neutralizing titers against the different animal sera tested. These results from single and multiple substitutions in the S protein suggest that positions 484K and 452R are particularly important for evading neutralizing antibodies in human, cat, and rabbit sera. We also tested multiple substitutions in the RBD of the S protein of the Omicron VOC, including mutation -501Y, -484A, -417N, -446S, -440K, -493R, -477N, -478K, -498R. As expected, the neutralizing titers of the tested sera against this omicron-like pseudotyped virus were significantly lower than the parental virus (Table 1).

## DISCUSSION

Since the unexpected emergence of SARS-CoV-2 in human populations, extensive efforts have been directed toward both elucidating the risks associated with emerging virus variants and identifying susceptible animal species to better understand the zoonotic/reverse zoonotic implications of the pandemic. In our study, we used pseudotyped virus assays to elucidate the roles of ACE2 from various animal species, including humans, in viral entry, which is a central event determining host susceptibility to SARS-CoV-2 infection. Using the S protein from the ancestral prototype (parental) SARS-CoV-2 strain (Wuhan-Hu-1), we found that several animal ACE2 receptors can efficiently interact with SARS-CoV-2 S protein to allow virus entry into cells. The efficiencies of virus entry among animal ACE2 receptors tested are not remarkably different from that of human ACE2, except for mink ACE2, which was consistently associated with comparatively low virus entry efficacy. Many animal species have been reported to be susceptible to SARS-CoV-2 infection either in experimental studies or by natural infection, as evidenced by clinical disease, viral replication in the respiratory tract and other organs, viral shedding/transmission, or seroconversion; these include domestic and large captive cats, dogs, cattle, mink, ferrets, otters, fruit bats, nonhuman primates, New Zealand White rabbits, hamsters, deer mice, bushy-tailed woodrats, striped skunks, and white-tailed deer (46–50). Other animal species either have not been tested or showed no consistent evidence of active viral infection. Among them, cats and dogs have been of particular interest due to their proximity to humans. These companion animals can be infected by SARS-CoV-2 in natural and experimental settings and usually remain asymptomatic, although some develop mild respiratory disease (21, 51–53). Overall, our pseudotyped virus entry results are consistent with previous animal susceptibility studies with most of the animal ACE2 receptors (human, cat, dog, cattle, camel, hamster, rabbit, mink, and white-tailed deer) tested in this report for virus entry (46–50). Although there are currently no or few reports of natural or experimental infection in horses (54) and camels, there have been concerns that SARS-CoV-2 may infect these animals, based on predictions from structural *in silico* analyses or cell-to-cell fusion assays using pseudotyped virus (15–17). Our results regarding the horse and camel ACE2 receptors and pseudotyped viruses provide a further impetus to study viral susceptibility in these animal species; however, our structural modeling (Fig. 4) coupled with previous experimental evidence (55) indicates that the horse ACE2 Y41H substitution may confer resistance to RBD binding of both parental and mutated S proteins. Recent reports showed no evidence of virus replication in a horse experimentally infected with SARS-CoV-2 (54), although this requires further confirmation. An experimental infection study of cattle revealed that SARS-CoV-2 infection in this species may occur but does not appear to be robust, which seems to support the results of pseudotyped virus assays conducted by us and others (15, 56). Interestingly, mink ACE2 was predicted to have a weak interaction with S protein in a previous *in silico* analysis study (17); similarly, our pseudotyped virus entry assay showed that mink ACE2 allowed viral entry, although at a relatively lower level than that observed with ACE2 from other animals or humans. This is somewhat surprising because mink are highly susceptible to SARS-CoV-2 infection, leading to a significant number of outbreaks of COVID-19 in mink farms with high morbidity/mortality (7, 8). It is likely that an unknown disparity exists between virus entry mediated by pseudotyped viruses and native cell-virus interaction for mink. Structural models were generated to gain insight into the interaction between the S protein and selected animal ACE2 with a focus on the residues interacting with K417, E484, and N501 on the S protein (Fig. 4). The respective ACE2 residues are mostly conserved with minor variations among human and animal ACE2s, which is in line with the pseudotyped virus assay results obtained in this study.

We also examined the effects of various mutations (417N, 452R, 478K, 484K, and 501Y) in the RBD, found in the Alpha (614G-501Y), Beta (614G-501Y-484K-417N), or Delta variants (452R-478K or 452R-484K), on virus entry in cells expressing human or animal ACE2 receptors using pseudotyped viruses. SARS-CoV-2 variants carrying 614G have replaced the prototype 614D virus and now are part of all major variants (57, 58), most likely because 614G is associated with enhanced fitness in susceptible cells, including human airway cells (58, 59). The 614G virus was also shown to enhance replication in the upper respiratory tract and transmission in infected hamsters (59, 60), although this was not observed in hACE2 transgenic mice (59). In human ACE2-expressing 293T cells, pseudotyped viruses carrying 614G alone have been reported to either increase (58, 61–64) or cause no change (36) in viral cell entry. In contrast to previous findings showing an increase in 614G cell entry in cells expressing human, cat, or dog ACE2 orthologs (58), pseudoviruses carrying the 614G mutation alone consistently showed decreased cell entry across all species in our assays. Structural studies have indicated that 614G does not result in a higher affinity toward ACE2 but instead results in allosteric changes conducive toward a more open conformation of the RBD in which it is better positioned to interact with the ACE2 receptor (58). The entry efficiency of the 484K single mutation alone has not yet been well studied. In our study using human ACE2-expressing cells, entry of the 614G or 484K mutant pseudotyped viruses was significantly decreased compared to the parental virus. In contrast, the 614G-501Y-484K (found in the Beta VOC) and 614G-501Y-484K-417N (found in Beta and Gamma VOCs) mutations in the S protein increased virus entry compared to the parental pseudotyped virus. In a previous report (56), pseudoviruses with these mutations did not change virus entry in cells expressing human and various animal ACE2 receptors, with the exception of murine ACE2-expressing cells (56). This observed difference in virus entry may be due to the different assay system, including cell types, variance of assays, or other factors.

Our structural modeling showed potential changes in the interactions of mutant RBD amino acid substitutions 501Y-484K-417N and human ACE2, which may affect RBD binding (Fig. 4B). The structural modeling performed in this study is consistent with previous reports, indicating that the N501Y substitution increases the affinity for human ACE2 via the formation of new contacts with hACE2 residues Y41 and K353 and that the salt bridges between E484 and hACE2 K31 as well as K417 and hACE2 D30 are lost following substitution to 484K and 417N (Fig. 4B; 65–67). While many of the animal ACE2 sequences analyzed in this study (cat, cattle, hamster, and rabbit) contain ACE2-RBD interaction sites consistent with humans, there are amino acid differences in the ACE2 receptors of dogs, mink, horses, and camels that could have significant effects on RBD-ACE2 interaction, especially when subjected to VOCs. The H33Y/H34Y ACE2 substitution found in dog and mink, respectively (Fig. 4C), was previously predicted to decrease RBD interaction, although several additional species with known susceptibility carry this substitution (e.g., cougar, lion, and tiger) (68, 69). Our modeling analysis suggests that the dog/mink Y33/34 residue in ACE2 could potentially increase RBD-ACE2 affinity when the RBD K417N substitution is present; however, the K417N substitution did not significantly increase pseudotyped virus entry in cells expressing ACE2 from dog and mink. This inconsistency may limit the significance of the H33/H34 residue of ACE2 and highlights the importance of wet lab research to supplement *in silico* predictions. Horse ACE2 contains a Y41H substitution, an amino acid that was shown by prior mutagenesis studies to abolish RBD binding (55, 70). This contrasts with our results indicating that horse ACE2 with 41H can facilitate viral entry, albeit at a lower level than ACE2 receptors with 41Y. Notably, variant-associated S proteins did not increase cellular entry mediated by horse ACE2, indicating an overall deficiency of S binding for this species. Lastly, the camel ACE2 contains a K31E substitution that was previously shown to abolish ACE2 binding (70); however, we observed robust viral entry in cells expressing the camel ACE2 receptor. Moreover, our structural predictions indicate that the K31E substitution in camel ACE2 may provide a basis for hydrogen bonding with 484K in the S variant. This is consistent with an increase in virus cell entry observed in camel ACE2-expressing cells following the E484K substitution, indicating that variants containing 484K may have increased susceptibility in camels. Overall, these structural insights provide some basis for understanding the ramifications of SARS-CoV-2 S mutations in emerging variants, although further structural and functional studies are required to confirm their effects in various animal species.

Our replication kinetics study using parental (lineage A), B.1, Alpha (B.1.1.7), and Beta (B.1.351) SARS-CoV-2 variants in CRFK cells stably expressing human ACE2 or hamster ACE2 revealed a comparatively higher replicative capacity of the Beta variant in these cells. This is consistent with the report of increased replication efficiency of the Beta variant over the Alpha variant in Vero cells (71). In our experimental infection study using hamsters infected with parental (lineage A) as well as Alpha (B.1.1.7) and Beta (B.1.351) VOCs, virus shedding among these viruses were comparable at 5 days postinfection (dpi) in nasal washes. However, more robust viral shedding was observed with the Beta VOC than the prototype parental virus or the Alpha VOC at 3 dpi, eventhough the same inoculum dose was used for all three viruses. This suggests that the Beta variant may replicate more efficiently than the other tested viruses in susceptible nasal epithelial cells during the early phase of infection, which would be conducive toward increased virus shedding and transmission during that period. The Alpha VOC appears to be transmitted highly efficiently (72, 73), and the Beta VOC has spread rapidly worldwide, which suggests that the Beta variant seems to have a replicative advantage over other previously circulating variants, although more data are required to confirm this (74).

The emerging VOCs have been associated with an adverse impact on antibody neutralization capacity by convalescent and immunized humans, which may affect vaccine- and infection-induced protection, risks of reinfection, and the potency of therapeutic monoclonal antibodies (MAbs) (75, 76). The full set of signature mutations on and around the RBD of the S protein, as well as mutations in other genomic regions of SARS-CoV-2 VOCs, contribute to the characteristics of each variant. Particular attention has been directed toward the roles of amino acid residues located on the S protein’s RBD due to its direct interaction with ACE2. To understand the effects of key mutations of the Alpha, Beta, Delta, and Omicron VOCs, we tested respective pseudotyped viruses in virus neutralization assays with convalescent human and cat (from a challenge study with the SARS-CoV-2 USA-WA1/2020 strain [77]) sera and hyperimmune rabbit sera (immunized with baculovirus-expressed prototype parental S protein). Substitution of N501Y is found in Alpha, Beta, and Gamma VOCs and is reported to have an increased binding efficacy to human ACE2 (78). Our result showed that the 501Y substitution did not significantly affect neutralization ability of all tested sera compared to the parental virus; this is consistent with a previous report whereby recombinant SARS-CoV-2 virus (within the genetic background of USA WA1/2020) carrying the 501Y or 501Y-614G mutations did not affect neutralization ability of BNT162b6 vaccine-induced antibodies in human sera (79). The 484K mutation has emerged independently in multiple variants and confers resistance to some MAbs targeting the S protein receptor-binding motif region (80, 81). Adding the 484K mutation into pseudotyped viruses carrying the full set of the Alpha variant-associated mutations on the S protein led to a considerable loss in neutralizing activity of antibodies in BNT162b6-elicited or convalescent human sera (82); this is in line with our results using pseudotyped viruses carrying the 484K or 484K-501Y mutations. Likewise, combined S protein substitutions, such as 484K-614G, 484K-501Y-614G, or 417N-484K-501Y-614G, reduced the neutralization ability of BNT162b6 vaccine-induced human sera and several therapeutic MAbs to mutated recombinant viruses in the genetic background of USA WA1/2020 (79); similarly, it affected the neutralization ability of convalescent human sera to pseudotyped viruses carrying 417N-484K-501Y-614G as shown here. The substitutions 452R and 478K are found in the Delta variant and other variants, such as the Epsilon variant, and have been on the rise in the United States and Mexico since early 2021 (83). Due to its relatively recent appearance, the impact of the 452R and 478K substitutions have not yet been well studied; they have been predicted to negatively affect human ACE2 binding, and were shown to reduce the neutralizing activity of antibodies from convalescent or vaccinated individuals (61, 84, 85). In our pseudovirus assay, we observed a significant reduction of neutralization activity of the tested sera with the pseudovirus carrying 452R but not 478K. The negative effect of 452R on neutralization is also seen with the 452R-478K double mutation, which significantly reduced neutralization activity of the sera. Interestingly, the neutralizing activities of sera with 452R-484K, 452R-478K, 501Y-484K, and 501Y-484K-417N pseudoviruses were comparably reduced in our assays. The negative impact of the 501Y-484K-417N-carrying pseudovirus on neutralizing antibodies elicited with mRNA-based vaccines was also observed by others (86). The recently emerged Omicron VOC has largely replaced the previous variants worldwide (18, 29) and carries a large number of substitutions in the S protein, including A67V, Δ69 to 70, T95I, G142D, Δ143 to 145, Δ211, L212I, insertion 214EPE, G339D, S371L, S373P, S375F, K417N, N440K, G446S, S477N, T478K, E484A, Q493R, G496S, Q498R, N501Y, Y505H, T547K, D614G, H655Y, N679K, P681H, N764K, D796Y, N856K, Q954H, N969K, and L981F (18, 29). The Omicron variant has been reported to evade neutralizing activity of most therapeutic monoclonal antibodies and has decreased neutralizing titers against convalescent patient sera up to 4- to 8-fold compared to the parental virus (87–89). Omicron’s evasion of neutralizing antibodies was also observed in our neutralization assays using the pseudotyped virus carrying 501Y-484A-417N-446S-440K-477N-478K-493R-498R substitutions against human and animal sera. The neutralizing titers against the Omicron variant were significantly reduced compared to the pseudovirus carrying the parental S (Table 1), with a 3- to 5-fold reduction in neutralizing titers, which is consistent with previous reports (87, 89).

In summary, our results obtained from a lentivirus-based pseudovirus system and hamster infection studies showed that a wide range of animal ACE2s support pseudotyped virus entry, and the key mutations found in the VOCs affect pseudotyped virus entry in cells expressing human or animal ACE2 as well as neutralizing activity of sera from humans, cats, and rabbits. The hamster infection study suggest a replicative advantage of the Beta variant over the parental and Alpha variant. The findings of this study highlight the importance of elucidating the roles of S mutations in detail and monitoring for evolving SARS-CoV-2 variants to assess their public health implications.

## MATERIALS AND METHODS

### Animal care and ethics statement.

All animal experiments were conducted in animal biosafety level 3 (BSL3) facilities at the Biosecurity Research Institute at Kansas State University according to protocols approved by the Institutional Animal Care and Use Committee at Kansas State University and the guidelines set by the Association for the Assessment and Accreditation of Laboratory Animal Care and the U.S. Department of Agriculture.

### Cells and plasmids.

HEK293, Crandell-Rees feline kidney (CRFK), and Calu-3 cells were purchased from American Type Culture Collection (ATCC; Manassas, VA). Vero E6 cells expressing human TMPRSS2 (Vero-TMPRSS2) were obtained from Creative Biogene (Shirley, NY) (90). Cells were maintained with either Dulbecco’s modified Eagle medium (DMEM) or Eagle’s minimal essential medium (MEM), both supplemented with 5% fetal bovine serum (FBS), 100 U/mL penicillin, and 100 μg/mL streptomycin. The codon-optimized cDNAs of the open reading frame (ORF) of the human or animal ACE2 gene with FLAG tag were synthesized by Integrated DNA Technologies (Coralville, IA) and cloned into pIRES-Neo3 (TaKaRa Bio, Mountain View, CA). For the ACE2 gene of white-tailed deer, because only a partial ORF is available, the full ORF was constructed with the human ACE2 gene. These plasmids were then designated pIRES-Neo-(species) ACE2-FLAG. The animal species from which ACE2 gene sequences (listed in Table S1 in the supplemental material) were derived are cat, dog, Arabian camel, European mink, horse, rabbit, cattle, Syrian golden hamster, and white-tailed deer. Pseudotyped viruses expressing SARS-CoV-2 S protein were generated by synthesizing the S gene, which was truncated by 26 amino acids at the C terminus, fused with a hemagglutinin (HA) tag by Integrated DNA Technologies, and cloned into plasmid pAbVec1 (Addgene, Watertown, MA), and designated pAbVec-SARS2-S. The parental S gene sequence was the prototype SARS-CoV-2 S gene from Wuhan (GenBank ID YP_009724390.1). This clone was then used to generate single or multiple mutations in the RBD of the S gene with a site-directed mutagenesis kit (Agilent, Santa Clara, CA) using primers listed in Table S2 and designated pAbVec-SARS2-S (mutant). Single mutations in the RBD include N501Y (Alpha variant), E484K, K417N, T478K, and L452R, and multiple mutations include N501Y + E484K (Gamma variant), L452R + E484K (Delta variant), L478K + L452R (Delta variant), N501Y + E484K + K417N (Beta variant), D614G + N501Y + E484K + K417N (Beta variant), and D614G + N501Y + E484A + K417N + G446S + N440K + S477N + T478K + Q493R + Q498R (Omicron variant). Each mutation was confirmed by Sanger sequencing analysis.

### Anti-SARS-CoV-2 antibodies from humans, cats, and rabbits.

Convalescent sera (Lotus 11 and 25) from COVID-19 patients were obtained from Thomas Rogers from the Scripps Research Institute, San Diego, CA, USA. Cat sera (Cat 247 and 903) were collected from cats enrolled in SARS-CoV-2 reinfection studies (91). Hyperimmune rabbit sera (Rabbit 4A and 7A) were obtained by immunizing rabbits with SARS-CoV-2 recombinant baculovirus-expressed S proteins based on the prototype Wuhan isolate. Negative sera from each species were also included in the study.

### Generation of CRFK cells stably expressing human or animal ACE2.

CRFK cells, plated the previous day, were transfected with pIRES-Neo-human (or cat, dog, cattle, horse, camel, hamster, rabbit, mink, or white-tailed deer) ACE2-FLAG. The transfected cells were then subsequently selected in the presence of 1 mg/mL G418. Expression of the ACE2 receptor of each animal species in the cells was confirmed by Western blotting using antibody against human ACE2 (Abcam, Waltham, MA). Parental CRFK cells served as a control (mock).

### Generation of SARS-CoV-2 S pseudotyped viruses.

The second-generation lentiviral packaging plasmid psPAX2 (Addgene), a reporter plasmid pUCGFP-Luc (Addgene), and parental or mutant pAbVec-SARS2-S were transfected into HEK293 cells to produce pseudotyped viruses. Briefly, cells plated in 6-well plates the previous day were transfected with three plasmids (1 μg each per well) using Lipofectamine 2000 (Thermo Fisher, Waltham, MA). Following overnight incubation, medium was replaced with fresh medium containing 5% FBS, and the cells were further incubated for 48 h. Supernatants were collected, and cell debris was removed by centrifugation at 400 × *g* for 10 min. Quantitation of pseudotyped viruses was performed using an HIV p24 assay kit (TaKaRa Bio) or ELISA for SARS-CoV-2 S (Sino Biological, Wayne, PA) before storing at –80°C.

### Pseudotyped virus entry assays.

To study the entry efficiency of parental or mutant S in cells expressing human or animal ACE2, HEK293 cells or CRFK cells expressing human or animal ACE2 were infected with pseudotyped virus carrying parental or mutant S protein. Briefly, cells plated the previous day were infected with each pseudotyped virus at a multiplicity of infection (MOI) of approximately 1 based on the p24 ELISA for pseudotyped virus preparation. Cell lysates were prepared at 48 h after infection, and firefly luciferase activity was measured on a luminometer (GloMax 20/20, Promega, Madison, WI). Fold change over the parental pseudotyped viruses was calculated for each mutant pseudotyped virus.

### Replication kinetics of SARS-CoV-2 variant strains in cells.

SARS-CoV-2 strains USA/WA1/2020 (lineage A, WA-A), USA/NY-PV08410/2020 (lineage B.1, NY-B.1), USA/CA_CDC_5574/2020 (lineage B.1.1.7, CA-B.1.1.7; Alpha variant), and South Africa (SA)/KRISP-K005325/2020 (lineage B.1.351, SA-B.1.351; Beta variant) were acquired from BEI Resources (Manassas, VA, USA; Table S3). Virus stocks were prepared by passaging on either Vero-TMPRSS2 (WA-A, NY-B.1, CA-B.1.1.7, and SA-B.1.351) or Calu3 cells (SA-B.1.351), and titers were determined using Vero-TMPRSS2 cells for 50% tissue culture infectious dose per mL (TCID_50_/mL), calculated using the Spearman-Karber method. Virus stocks were sequenced by next-generation sequencing (NGS) using the Illumina MiSeq. All variant stock viruses were in consensus with the original sequenced strains in GenBank. A mutation (PWRAR) in the S furin cleavage site of the SA/KRISP-K005325/2020 Vero-TMPRSS2 passage 1 stock virus was detected; this stock was used for inoculation of hamsters. The SA/KRISP-K005325/2020 stock was subsequently passaged on Calu3 cells, and NGS results showed that this stock contained only 13% of the furin site mutation (PWRAR); this stock was used for the *in vitro* virus replication kinetic experiments.

### SARS-CoV-2 variant replication kinetics in human and hamster ACE2-expressing CRFK cells.

SARS-CoV-2 variant replication kinetics were performed with CRFK-human ACE2 or CRFK-hamster ACE2 cells. Cells were inoculated with approximately 0.01 MOI of each virus strain except for B.1.351 which was inoculated at approximately 0.001 MOI, and cell culture supernatants were collected at 24, 48, and 72 h postinfection (hpi). Inoculum (defined as 0 hpi) and the time point-collected supernatants were then titrated on Vero-TMPRSS2 cells.

### Infection of SARS-CoV-2 variants in the hamster model.

Hamsters (lineage A, *n = *8; B.1.1.7, *n = *4; B.1.351, *n = *8) were inoculated intranasally with 1 × 10^5^ TCID_50_/mL of virus in 0.1 mL of DMEM. Half of the hamsters were humanely euthanized and necropsied at 3 days postchallenge (dpc) and the remaining at 5 dpc. Nasal washes were collected from all hamsters at both 3 and 5 dpc, and lungs were collected at necropsy. Nasal wash samples were vortexed and stored at –80°C until analysis. Lung homogenates were prepared by homogenizing 200 mg of tissue in a tube containing 1 mL of DMEM and a steel bead with a TissueLyser LT (Qiagen, Germantown, MD, USA) for 30 s at 30 Hz repeated 3 times. Nasal washes and lung homogenates were filtered through a 0.2-μm filter before virus titration on Vero-TMPRSS2 cells.

### Neutralization assay of convalescent or virus-infected sera from humans, cats, or rabbits against pseudotyped viruses expressing SARS-CoV-2 parental or mutant S proteins.

The effects of the mutations in S on antibody-neutralizing activity were examined with a panel of SARS-CoV-2 serum samples from humans, cats, and rabbits and pseudotyped viruses carrying parental and mutant SARS-CoV-2 S. Serial 2-fold dilutions starting at 1:12.5, 1:25, or 1:50 of each heat-inactivated serum sample were mixed with a constant amount of each of the pseudotyped viruses carrying parental or mutant S and incubated for 1 h at 37°C. Then, the serum-pseudotyped virus mixture was transduced onto CRFK-human ACE2 cells. Cell lysates were prepared at 48 h after transduction, and the relative luminescent units (RLU) were measured. Inhibition curves with each serially diluted serum sample were generated, and the neutralizing titers were calculated as the reciprocal of the serum dilution that showed 50% reduction by using GraphPad Prism software version 6 (San Diego, CA).

### Structural modeling of ACE2-RBD interactions.

Protein structural models were produced for the ACE2 interaction with the S protein receptor-binding domain (RDB) using the ACE2 sequences for each species listed in Table S1 using SWISS-MODEL (92). The Protein Data Bank (PDB) entry 6LZG (93) was used as a template for model building. The ACE2 sequences for each species, corresponding to residues found in the 6LZG template, were used, and either the parental SARS-CoV-2 RBD sequence or the mutated sequence (N501Y-K417N-E484K) was added as a hetero target. Structures were analyzed and images were created using PyMOL (94).

### Statistical analysis.

Statistical analysis was performed using GraphPad Prism software version 6 (San Diego, CA). A one-way analysis of variance (ANOVA) followed by a Tukey *post hoc* test on the log_10_-transformed firefly luminescent units or neutralization titers was used to compare the parental and mutant pseudotyped viruses. To identify significant differences between ACE2-expressing cell cultures or hamsters infected with the different SARS-CoV-2 strains, virus titer data were first log_10_ transformed, and row means and standard deviations were calculated. The data were then analyzed by two-way ANOVA, followed by a Tukey’s multiple-comparison test; statistical differences are indicated with an asterisk (*) representing a *P* value of <0.05. Data are representative of at least two independent experiments. To identify significant differences between groups of hamsters challenged with the different SARS-CoV-2 strains, virus titer data were first log_10_ transformed, and row means and standard deviations were calculated. The data were then analyzed by two-way ANOVA followed by a Tukey’s multiple-comparison test, and statistical differences are indicated with an asterisk (*) representing a *P* value of <0.05. Data are representative of at least two independent experiments.

### Data availability.

Data will be made available upon request.
