# Supplementary material for: Effects of Spike Mutations in SARS-CoV-2 Variants of Concern on Human or Animal ACE2-Mediated Virus Entry and Neutralization
Source: Microbiol Spectr. 2022 May 31;10(3):e01789-21. doi: 10.1128/spectrum.01789-21 (PMC9241865; doi:10.1128/spectrum.01789-21)
Supplement: SUPPLEMENTAL FILE 1 — Supplemental material. Download spectrum.01789-21-s001.pdf, PDF file, 0.2 MB [file spectrum.01789-21-s001.pdf]

**Supplemental Table 1. Sources of human and animal ACE2 gene sequences.**

| **Species** | **Scientific name** | **Source** |
|---|---|---|
| Human | Homo sapiens | NP_068576.1 |
| Cat | Felis catus | NP_001034545.1 |
| Dog | Canis lupus | NP_001158732.1 |
| Cattle | Bos taurus | NP_001019673.2 |
| Horse | Equus caballus | XP_001490241.1 |
| Camel – dromedary | Camelus dromedarius | XP_010991717.1 |
| Hamster | Mesocricetus auratus | XP_005074266.1 |
| Rabbit | Oryctolagus cuniculus | XP_002719891.1 |
| European mink | Mustela lutreola biedermanni | MT560518 |
| White tailed deer | Odocoileus virginianus | XP_020768965.1 |

**Supplementary Table 2. Primers used for mutagenesis of the SARS-CoV-2 S gene.**

| Name | Sequences |
|---|---|
| SA2-D614G-F | 5-TTGCTGTTCTTTATCAGGGTGTTAACTGCACAGAAGT-3 |
| SA2-D614G-R | 5-ACTTCTGTGCAGTTAACACCCTGATAAAGAACAGCAA-3 |
| SA2-N501Y-F | 5- GGTTTCCAACCCACTTATGGTGTTGGTTACCAAC-3 |
| SA2-N501Y-R | 5- GTTGGTAACCAACACCATAAGTGGGTTGGAAACC-3 |
| SA2-E484K-F | 5- CCTTGTAATGGTGTTAAAGGTTTTAATTGTTAC-3 |
| SA2-E484K-R | 5- GTAACAATTAAAACCTTTAACACCATTACAAGG-3 |
| SA2-K417N-F | 5- CCAGGGCAAACTGGAAACATTGCTGATTATAAT-3 |
| SA2-K417N-R | 5- ATTATAATCAGCAATGTTTCCAGTTTGCCCTGG3- |
| SA2-T478K-F | 5' – ATCTATCAGGCCGGTAGCAAACCTTGTAATGGTGT-3 |
| SA2-T478K-R | 5' – ACACCATTACAAGGTTTGCTACCGGCCTGATAGAT-3 |
| SA2-L452R-F | 5-GGTTGGTGGTAATTATAATTACCGGTATAGATTGTTTAGGAAGTCTA-3 |
| SA2-L452R-F | 5-TAGACTTCCTAAACAATCTATACCGGTAATTATAATTACCACCAACC-3 |

**Supplementary Table 3. SARS-CoV-2 strains used for *in vitro* replication kinetics and hamster infection experiments.**

| Strain | BEI number | Lineage | Clade | Spike Mutations | Variant |
|---|---|---|---|---|---|
| USA/WA1/2020 | NR-52281 | A | S | na | |
| USA/NY- PV08410/2020 | NR-53514 | B.1 | GH | D614G | B.1 |
| USA/CA CA_CDC_5574/2020 | NR-54011 | B.1.1.7 | GR | A570D, D614G, D1118H, H69del, N501Y, P681H, S982A, T716I, V70del, Y145del | Alpha |
| South Africa/KRISP-K005325/2020 | NR-54009 | B.1.351 | GH | A243del, A701V, D80A, D215G, D614G, E484K, K417N, L18F, L242del, L244del, N501Y | Beta |

na=not applicable
